# Immediate Implantation with Autologous Mineralized Dentin Graft versus Deproteinized Bovine Bone as Space-Filling Substitute in Maxillary Anterior Zone: Retrospective Radiological and Clinical Study

**DOI:** 10.3390/jcm13185521

**Published:** 2024-09-18

**Authors:** Ihsan Caglar Cinar, Mohammed Zboun, Alper Saglanmak, Eitan Mijiritsky

**Affiliations:** 1 Department of Oral Implantology, Faculty of Dentistry, Istanbul University, Fatih, Istanbul 34093, Türkiye; alper.saglanmak@istanbul.edu.tr; 2 Department of Oral & Maxillofacial Surgery and Periodontology, Faculty of Dentistry, Arab American University, 13 Zababdeh, Jenin 240, Palestine; mohammed.alzboun@aaup.edu; 3 Department of Head and Neck Surgery and Maxillofacial Surgery, Tel-Aviv Sourasky Medical Center, School of Medicine, Tel Aviv University, Tel Aviv 64239, Israel; mijiritsky@bezeqint.net

**Keywords:** immediate implantation, autogenous mineralized dentin graft, deproteinized bovine bone, tooth extraction

## Abstract

**Background/Objectives**: Various bone substitutes have been recommended to augment the horizontal gap following immediate implantation. The purpose of this study was to compare the effectiveness of an autogenous mineralized dentin graft or a deproteinized bovine bone in horizontal gap augmentation following immediate implant placement in the maxillary anterior region. **Methods**: A total of 110 patients underwent tooth extraction followed by immediate implant placement. The patients were divided into two groups. The first group received an autogenous mineralized dentin graft (the test group) while the second group received a deproteinized bovine bone (the control group) to augment the horizontal gap. Preoperative (T0), immediate postoperative (T1), and 1-year postoperative (T2) cone beam computed tomography scans were taken from all the patients. Linear measurements were recorded 1 mm (R1) and 5 mm (R2) points apical to the implant platform at both T1 and T2 time intervals. Pink Esthetic Scores and prosthetic complications were evaluated as well. **Results**: There were 57 patients with a mean age of 45.42 ± 9.86 (range 24–63 years) selected as the test group and 53 patients with a mean age of 40.28 ± 11.69 (range 20–63 years) as the control group. The mean reduction in the buccal bone plate at R1 was 6.39 ± 3.78% in the test group and 6.99 ± 5.01% in the control group (*p* > 0.05). The mean reduction in the buccal bone plate at R2 was 5.46 ± 4.98% in the test group and 6.77 ± 7.60% in the control group (*p* < 0.05). The PES and prosthetic-related complications were shown to be negligible between the groups (*p* > 0.05). **Conclusions**: The efficiency of using an autogenous mineralized dentin graft for horizontal gap augmentation showed similar results in comparison to using a deproteinized bovine bone in relation to buccolingual socket reduction following immediate implantation.

## 1. Introduction

Dental implantation is considered the treatment of choice for missing teeth replacement that leads to oral and dental function and aesthetic restoration. However, osseous defects such as alveolar bone loss due to improper healing or wound management following dental extraction or tooth loss prompt a challenging modality of treatment [1].

Several methods were introduced to reduce such bone resorption [2,3]. Immediate implantation in the former extraction socket is considered one of these methods. It possesses many advantages over the others, such as avoiding additional surgeries, shortening treatment time, and reducing the period of edentulism [4]. The rationale behind immediate implantation is the integrity of the dental implant fixture into the fresh socket walls, which can minimize bone resorption [5]. However, among all the perioperative complications and difficulties that clinicians may encounter during the immediate implantation procedure, there are two common challenges which are considered hard to cope with and may affect the first stage of the healing process. The first is the horizontal distance between the buccal bone plate and the implant surface (the horizontal gap), which is created by the discrepancy between the socket (larger) and implant (smaller) diameters, while the second is the primary wound closure of the surgical site created by tooth removal [6].

The horizontal gap exists due to the erratic morphology of the extracted tooth socket, where the implant surface and the platform periphery in particular may not fit securely against the inner buccal alveolar bone walls after implant insertion. It is necessary to understand the biological capability of the body to fill the existing space with newly formed bone and the modality of treatment to establish a successful aesthetic and function outcome of the dental implant. A horizontal gap > 2 mm results in inadequate bone formation and consequently increases the potential of implant body exposure followed by aesthetic failure [7,8].

Introducing autologous particulate dentin as a bone substitute gained attention in the 1980s–1990s. The growing interest in such a graft emerged after the finding of bone morphogenetic proteins (BMPs) in dentin that provide osteoinductive properties, which in turn can promote bone marrow mesenchymal stem cell differentiation and accelerate osteogenesis. Dentin particles can be used as a graft material due to their similarity to a high degree with bone composition [9].

In relation to xenografts, an organic deproteinized bovine bone is the most commonly used bone graft, and there are plentiful studies which proved their osteoconductivity with a low biodegradation rate and tissue reaction. However, bovine-derived graft predilection for prion disease transmission was reported [10]. An autogenous mineralized dentin graft (AMDG) can be differentiated from the xenogeneic bone graft by the presence of BMPs as they were detected in the dentin. Their ability to induce de novo bone formation at orthotopic and heterotopic sites was also observed [11,12]. Furthermore, using dentin particulate eliminates the possibility of such infectious disease transmission, and it is considered less expensive in spite of the preparation period and techniques needed to make it ready for application.

The harmony of prosthetic restoration with the soft tissue is deemed a pivotal point for the patient’s esthetic outcome. The so-called pink esthetic, which refers to the esthetic of the soft tissue surrounding the implant-supported restoration, is associated with the proper management of the soft tissue on one hand and the stability of the alveolar bone beneath it on the other hand. For such drawbacks that may influence the soft tissue’s final esthetic outcome, the “Pink Esthetic Score” (PES) was introduced as an index to evaluate various characteristics of the soft tissue around single-tooth implant prostheses [13]. 

Although there are plenty of preclinical and clinical studies on AMDGs in the literature focused on socket preservation and sinus augmentation, there are still no sufficient studies on AMDG application with immediate implantation. The objectives of the present study were to primarily evaluate the dimensional changes in the buccal bone plate after immediate implantation using different graft materials (an AMDG and Bio-Oss^®^) in the aesthetic zone with a horizontal gap exceeding 2 mm. Secondarily, the PES and prosthetic complications will be examined as well.

## 2. Materials and Methods

### 2.1. Population and Study Design

This retrospective study was designed to investigate the remodeling course of the buccal alveolar bone in 110 patients after a surgical intervention protocol represented by a single or multiple dental extraction followed by immediate implantation in the maxilla (from the second premolar [left] to the second premolar [right]). The gap created as a result of the discrepancy between the implant and the socket diameters was filled with a specific bone substitute as required, where an AMDG was used in 57 patients while a deproteinized bovine bone (Bio-Oss^®^, Geistlich Pharma AG, Wolhusen, Switzerland) was used in 53 patients. 

The inclusion criteria for the selected patients were to have been indicated for dental extraction due to endodontic failure, root fracture, an untreatable carious lesion, or endodontic–periodontal complications; a horizontal gap > 2 mm after implant placement; and the existence of an integral and sound buccal bone plate with a thickness ≥ 1 mm to surround the immediate dental fixture in the anterior maxilla. The exclusion criteria taken into consideration were as follows: a horizontal gap diameter < 2 mm or >4 mm; acute infection located in the implant site; active periodontal disease; smokers with consumption that exceeded 20 cigarettes per day; excessive consumption of alcohol; the presence of systemic or local diseases that may interfere with bone repair (including diabetes, hypothyroidism, hyperparathyroidism, osteoporosis, and osteoarthritis); patients receiving chemotherapy and/or radiation therapy in the previous 5 years; immunocompromised patients; patients administering medications that may prohibit implant placement or may interfere with osseointegration or gingival tissue healing (including bisphosphonates, corticosteroids, etc.); patients with pregnancy; and patients unable or too undisciplined to attend follow-ups.

All patients were informed about the possible complications and the potential risks in addition to the feasible benefits of the treatment modality. All information was provided in written and oral form, and the patients’ consent forms were signed. Accordingly, the surgical procedure was performed with respect to the Declaration of Helsinki. The procedure protocol was approved by the Ethics Committee of the Faculty of Dentistry, Istanbul University, under the registration number (2024/45).

### 2.2. Surgical Procedure

The patients were administered a 2 g oral tablet of amoxicillin one hour before surgery and rinsed with 0.2% chlorhexidine gluconate mouthwash as a prophylactic antibiotic regimen. All procedures were performed by only one surgeon (I.C.C.) in a period between May 2022 and November 2023 at the dental clinics of the hospital of Istanbul University, Istanbul, Türkiye. Initially, local anesthesia was administered using an Articaine solution containing 1:200,000 epinephrine (Ultracain-DS; Hoechst Marion Roussel, Istanbul, Türkiye). Thereafter, dental extraction of the indicated tooth/teeth occurred with periotomes and forceps. The highest attention was paid not to cause any trauma to the surrounding bone and vital structures, thus preserving the bony socket wall. Subsequently, meticulous debridement was carried out to remove any soft tissue attached to the bony site followed by exploring the integrity of the alveolar bone preceding the dental implantation using a probe (15 UNC Colour-Coded, Hu-Friedy, Chicago, IL, USA). Following the extraction procedure, a full-thickness flap was elevated using a 2.5 mm Lucas Curette (Helmut Zepf, Seit-ingen-Oberflacht, Germany). Any fracture-inducing buccal bone trauma attained during the extraction procedure was met with the patient file being excluded from the study.

The implant bed was prepared in the socket using dental implant surgical drills according to the manufacturer’s instructions under controlled torque and speed. The drilling was achieved to extend 3–4 mm beyond the removed tooth apex bed with the aim of yielding primary stability. The final osteotomy drill was used followed by the insertion of a sterile implant into the prepared osteotomy site using a manual implant ratchet. The dental implants were submerged 1–1.5 mm below the crestal margin of the buccal bone. The design of the two-piece dental implant used in the patients included in the present study was a conventional threaded tapered-screw-type titanium dental implant system (Detech, DE|Tech Implant Technology, Ankara, Türkiye). With respect to the implant dimensions, the dental fixtures used in the selected patients ranged between 3.5 and 3.8 mm in diameter and 10, 12, and 14 mm in length.; all the implants were sized according to the dimensions of the osteotomy site as long as the gap between the installed implant and the internal buccal socket wall was kept >2 mm and <4 mm. The width of the gap was measured after implant placement using a periodontal probe.

**Figure 1:**
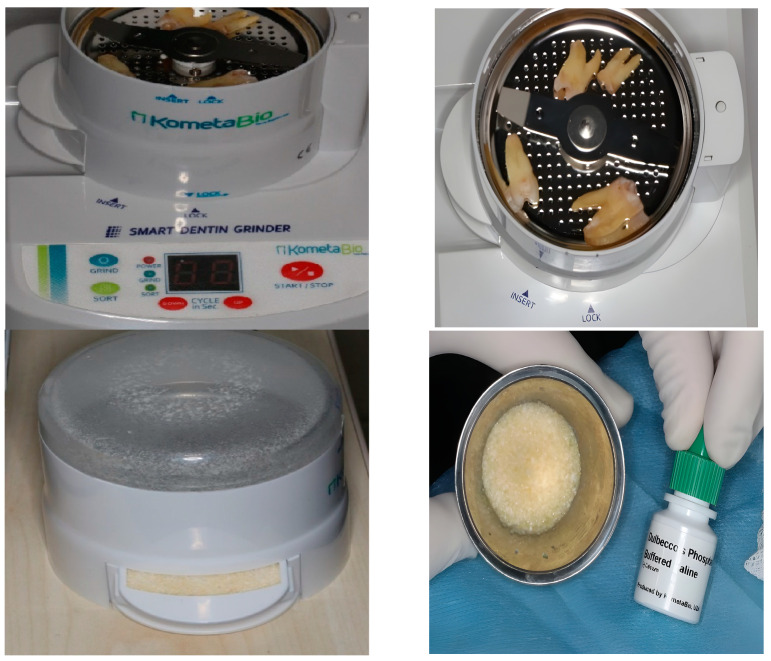
Preparation of autogenous mineralized dentin graft.

Two types of grafts were evaluated in this study as mentioned earlier. The AMDG was prepared according to the manufacturer’s instructions. The extracted teeth were treated by removing the enamel and cement layers with a dental turbine. The rest of the dentine structure was ground to certain particles, with their size ranging between 300 and 1200 µm, using a dentin grinder (Kometabio Smart Dentin Grinder, New York, NY, USA). The next step was to keep the particles in a dentin cleanser solution (Kometabio, New York, NY, USA) (20% ethanol + 80% sodium hypochlorite) for 10 min, which acts as a disinfectant agent. Then, the solution was detracted with gauze pads. The treatment of the particles proceeded by adding a phosphate-buffered saline (PBS) solution for 3 min to eliminate the remnant cleanser solution. Once again, the solution was discharged, making the mineralized dentin graft ready for use (Figure 1).

**Figure 2:**
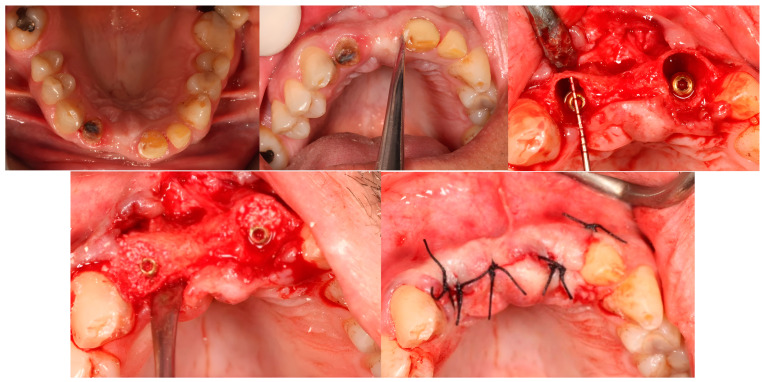
Immediate implantation and horizontal gap augmentation.

As the indicated number of implants were inserted, the gaps were filled with either the AMDG or Bio-Oss^®^ graft and then the flap was closed using a 3/0 silk suture (Silk, Dogsan, Istanbul, Türkiye) (Figure 2). The patients were kept on oral antibiotics (Augmentin-Bid, Glaxosmithkline, Istanbul, Türkiye), analgesics (Arveles, Ufsa, Istanbul, Türkiye), and mouthwash (Chlorobenzyl, Drogsan, Ankara, Türkiye) for 7 days. Ten days later, the sutures were removed. A clinical follow-up examination was conducted once a month, reminding the patients of the necessity of oral hygiene and to keep it maintained.

### 2.3. Radiographic Measurements

**Figure 3:**
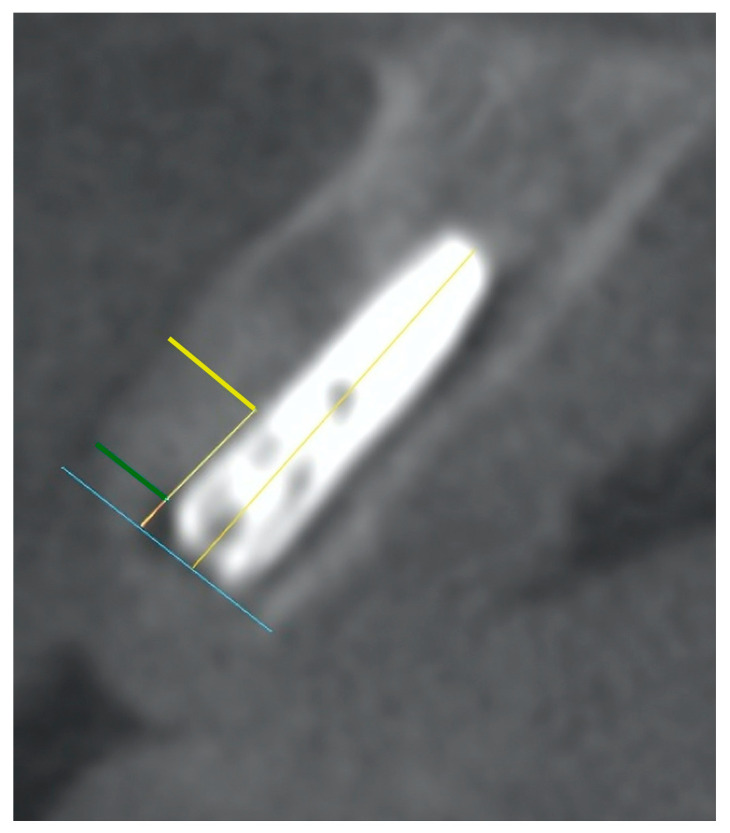
CBCT measurements and landmarks: blue line = implant collar reference; green line = R1, which is located 1 mm apical to the implant collar reference; yellow line = R2, which is located 5 mm apical to the implant collar reference.

Radiographic evaluation was achieved using CBCT shortly before extraction (T0), within 2 days after the surgical procedure (T1), and 1 year postoperatively (T2). All the scans were conducted at the same radiology center using the same parameters (110 kVp, 24 s, 5.7 mA, and voxel size 0.2 mm; field of view 6 cm × 8 cm). All the dimensions required for this study were collected, which were measured twice for certainty by the same radiologist specialist at 2 different time points. The following measurements were selected for evaluation: the thickness of the buccal bone plate, the width of the horizontal gap, and the distance from the buccal surface of the implant to the external aspect of the buccal bone together (the horizontal gap width in addition to the buccal bone plate thickness). Built on the transverse (axial) view, a reference was obtained by drawing a first line from the implant platform to the apex (parallel to the long axis of the implant); then, a second line perpendicular to the first one was drawn at the apex point. Based on the second line, two parallel lines were drawn; one was 1 mm apical (R1), and the other one was 5 mm apical to the implant collar (R2) (Figure 3).

### 2.4. Clinical Observation

The operation areas were classified as anterior (from the left canine to the right canine) and posterior (premolar regions). An evaluation of the esthetic outcome of the soft tissue around the implant-supported prosthesis was performed based on a PES assessment. Accordingly, frontal photographs were obtained and transferred into a PowerPoint presentation in a random order, while two experienced investigators who were uninformed of the methodology of the study reviewed and assessed all the images on the same computer. Seven variables were assessed, including the mesial papilla, distal papilla, soft tissue level, soft tissue contour, alveolar process deficiency, soft tissue color, and soft tissue texture. The point score given for each variable was from 0 = ‘very bad’ to 2 = ‘excellent’. The total score for each sample was registered as described with a maximum score of 14. Three weeks later, the investigators re-evaluated the images once again. Since a cement-retained system was chosen for all restorations, a prosthetic examination was carried out to record any complications, including abutment screw loosening, abutment screw fracture, ceramic chipping, and the decementation of implant-supported prostheses.

### 2.5. Statistical Analysis

The data were analyzed using the Statistical Package for the Social Sciences (SPSS) 26.0 statistics package program. Categorical data for the AMDG and Bio-Oss^®^ bone graft applications are given as numbers and percentages, and the numerical data are given as mean values and standard deviations. The suitability of the numerical variables of the patients who underwent AMDG and Bio-Oss^®^ bone grafting to normal distribution was determined by looking at the skewness and kurtosis values. It was observed that, except for the reduction percentages of the patients at 1 mm and 5 mm, all other numerical values followed the rules of normal distribution. The reference value taken for the normal distribution was within ±1.96. A Chi-square test was used to compare the gender, surgical areas, and presence of prosthetic complications of patients who underwent AMDG and Bio-Oss^®^ bone grafting. An Independent Sample *t*-test was used to compare the age, PES, (T0) buccal bone width at 1 mm and 5 mm, (T1) horizontal gap, and (T2) buccal bone width values of patients who underwent AMDG and Bio-Oss^®^ bone grafting. A Mann–Whitney U test was used to compare the reduction percentages of the patients. In addition, an Independent Sample *t*-test or Mann–Whitney U test was used to examine the relationships between parameters according to the dental areas of the patients. In the entire study, the values 0.05 and 0.01 were taken as levels of statistical significance.

## 3. Results

All the patients completed the observation period, and the implant survival rate was 100%. Fifty-seven patients who underwent the AMDG and fifty-three patients who underwent Bio-Oss^®^ bone graft augmentation were included in the present study. A significant difference was shown between the AMDG group, 45.42 ± 9.86, and the Bio-Oss^®^ bone graft group, 40.28 ± 11.69, with respect to age (*p* < 0.05). A comparison of the gender, age, and tooth area data of the patients with AMDG and Bio-Oss^®^ bone grafting is shown in Table 1.

The comparison of the buccal bone width, horizontal gap, prosthetic complications, and PES data of patients who received AMDG and Bio-Oss^®^ bone grafting is presented in Table 2.

No significant difference was reported between the T0 buccal bone width, T1 horizontal gap, T2 buccal bone width, and bone resorption amount up to 1 mm below the implant collar in patients who underwent AMDG and Bio-Oss^®^ bone grafting (*p* > 0.05).

Comparing the AMDG and Bio-Oss^®^ bone-grafted groups showed no significant difference with respect to the T1 horizontal gap values at 5 mm (*p* > 0.05). However, a significant difference was found between the T0 buccal bone width, T2 buccal bone width, and bone graft reduction amount at 5 mm (*p* < 0.05). According to these findings, the T0 buccal bone width and T2 buccal bone width values at 5 mm were higher in patients with the AMDG bone graft compared to those with the Bio-Oss^®^ bone graft. The graft reduction amount was significantly higher in the patients with the Bio-Oss^®^ bone graft compared to those of the patients with the AMDG bone graft (*p* < 0.05).

**Figure 4:**
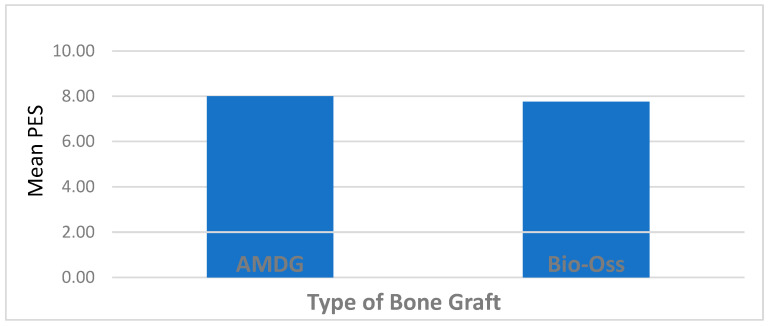
The mean PES scores of patients.

In contrast, no significant difference was found between the PES values of the AMDG and Bio-Oss^®^ bone-grafted groups (*p* > 0.05), (Figure 4).

A comparison of the buccal bone width, horizontal gap, prosthetic complications, and PES findings based on bone graft type and the region of operation are described in detail in Table 3.

There was no significant difference between the T1 horizontal gap, T2 buccal bone width, and PESs at 1 mm and 5 mm in the anterior region of both the AMDG and Bio-Oss^®^ groups (*p* > 0.05). However, there were significant differences between the T0 buccal bone width and reduction amount at 1 mm and 5 mm in the anterior region of the groups (*p* < 0.05). According to these data, the T0 buccal bone width values at 1 mm and 5 mm in the anterior regions of the AMDG group were significantly higher compared to the Bio-Oss^®^ group, though the reduction percentages were lower in the anterior tooth regions of the AMDG group compared to the Bio-Oss^®^ bone group. On the other hand, no significant difference was shown between the T0 buccal bone width, reduction amount, and PES measurements at 1 mm and 5 mm in the posterior region of patients with the AMDG and Bio-Oss^®^ bone grafts (*p* > 0.05). Nevertheless, a significant difference was found between the T1 horizontal gap and T2 buccal bone width measurements at 1 mm and 5 mm in the posterior region of the groups (*p* < 0.05). Moreover, the T1 horizontal gap and T2 buccal bone width values at 1 mm and 5 mm were higher in the AMDG group than in the Bio-Oss^®^ group.

The distribution of the prosthetic complication data was calculated and analyzed according to bone graft type and region of operation (Table 4).

The prosthetic complications in the anterior and posterior regions in the groups were deemed to be statistically comparable to one another (*p* > 0.05).

## 4. Discussion

An immediate implantation procedure directly after dental extraction is a common modality of treatment, which is well-documented in the literature. Numerous studies have shown comparable success rates whether implant placement was achieved immediately after dental extraction or after the site was healed [14,15]. Several studies were conducted to evaluate the influence of horizontal gap grafting on the esthetic of the area following immediate implantation in fresh sockets [16,17]. 

The main purpose of the present study was to evaluate the buccal bone thickness in the maxillary esthetic zone after successful immediate implantation with horizontal bone augmentation. According to the data obtained, this study is deemed one of the scarce human trials in terms of the utilization of autogenous mineralized dentin grafts following immediate implantation.

One of the potential difficulties that clinicians may perioperatively encounter is the disparity of diameters between the alveolar socket (larger) and the implant (smaller), creating a gap between their perimeters, which is defined as a “horizontal gap” [9]. Augmenting such a gap with a bone graft is still controversial. Some authors refute the effectiveness of any regenerative material applied into a gap > 2 mm as long as an intact buccal bone plate of a thickness > 1 mm exists [18]. In contrast, other authors state the necessity of grafting the larger buccal gaps after immediate implantation to enhance bone formation within such gaps and minimize the horizontal resorption of the buccal bone. It is noteworthy that the findings of the earlier studies may be attributed to the characteristics of the buccal bone included in their samples, since patients with buccal bone widths < 1 mm and/or bony fenestration were included in such studies [19]. Among the advantages of performing immediate implantation is the enhancement of esthetic outcomes. A systematic review of 50 studies involving 6 RCTs, 6 cohorts, 5 cross-sectional investigations, and 33 case series recorded acceptable aesthetic outcomes, though a potential risk of mid-facial mucosal recession was reported. Moreover, immediate implantation associated with bone-grafting procedures was documented in many studies showing enhanced esthetic outcomes as well [20].

As has been recognized, teeth and bone tissue have the same embryological origin as they are derived from the cells of the neural crest [21]. They also share many compositions in common such as organic and inorganic components and water and possess the properties of osteoconduction and osteoinduction. In autogenous dentin, hydroxyapatite adopted the form of calcium phosphate with low crystalline content, which facilitates its degradation with the help of osteoclasts. Despite its lack of osteogenic characteristics and recipient limitation, it still has favorable behavior and averts any possible complications and morbidities which might be experienced during graft-harvesting procedures. Several in vivo studies determined the osteoconductivity and osteoinductivity of autogenous dentin in addition to its integration and resorption capacity [22,23].

A dentin graft can be produced in a mineralized or demineralized form. The demineralization treatment brings about the advantage of revealing the collagen matrix and releasing growth factors, resulting in a rise in the regenerative capacity [24]. However, such a process has some drawbacks represented by a relative destruction of growth factors and a decrease in osteoconductivity. The mineralized form, on the other hand, preserves both the organic and inorganic components, and it can be prepared pre- or perioperatively in the clinic by any trained dental clinician or surgeon following dental extraction. Therefore, the extracted tooth, which formerly was known as a disused compromised organ, can be utilized as a low-cost bone substitute [25]. It is noteworthy that the treatment of the dentin particulate graft in the present study was constricted to the manufacturer’s instructions for the cleanser agent kit (Kometabio, New York, NY, USA). The purpose of using NaOCl is ascribed to the benefit of its disinfection properties, which reduces the potential tendency of inducing any kind of infection by any remnant bacteria in the prepared dentin graft. Although previous studies have used the same cleanser kit [26], such treatment still has an ambiguous effect on the structure and biology of dentine.

Autogenous-dentin-graft-related studies were scarce in the literature. In addition to that, only case reports and case-series articles were found with disparate methodologies [27,28]. Elfana et al. evaluated the outcomes of augmenting the extraction socket with mineralized and demineralized autogenous dentin followed by collagen membrane coverage. The radiographic assessment carried out 6 months postoperatively showed no significant difference between both grafts with respect to the changes recorded in the width of the alveolar bone and the heights of both the buccal and lingual plates of the alveolar bone [29]. The findings of the present study support Pang et al. in their comparison between an autogenous dentin graft and an organic bovine bone, which recorded favorable wound healing and new bone formation. It showed no statistically significant difference in the volume of the preserved bone after 6 months of bone graft augmentation, which is in accordance with our findings [30]. Pohl et al., in their microscopic examination, also observed new bone formation around the AMDG, which had been harvested from an impacted third molar and applied in the maxillary sinus [31]. 

After immediate implantation, the preservation of the bone from being resorbed might be influenced by multiple factors such as the size of the socket, thickness of the buccal bone plate, dimensions of the buccal gap, diameter of the implant, position of the implant, operation technique, utilization of bone grafts, and using a connective tissue graft [32]. Qahash et al. confirmed a high horizontal buccal bone reduction in animals with low buccal bone plate widths. An in vivo study reported greater horizontal buccal bone loss in patients with lower width values of the buccal bone plate [33]. Another study was conducted by Sanz et al. on 86 patients to evaluate the efficacy of deproteinized bovine bone mineral (DBBM) grafting in the horizontal gap after immediate implant placement in the anterior maxilla. The comparison was performed between a DBBM group and a control group, which received the same treatment with no grafting materials applied in the gap. The width of the buccal bone wall recorded in the grafted group was 0.94 mm, while it was 0.87 mm in the control group. A significant reduction in the horizontal crest bone dimension was noticed during a healing process over 4 months, particularly in the buccal aspect of the alveolar crest. Such a reduction was greater in the control group with a proportion of 38% (1.6 mm) while only 29% (1.1 mm) was recorded in the test group. In addition to that, there was a remarkably higher loss of bone thickness at the sites of the anterior maxilla, which already possessed a thinner buccal bone wall. Consequently, the augmentation of the gap with DBBM grafting material showed a significant drop in the horizontal bone changes in the buccal bone thickness, and more prominently in patients with a thinner buccal bone plate [19]. According to the data of the present study, it can be speculated that the reason for the high rate of resorption is the difference recorded in the initial bone thickness. The average value of the initial bone thickness in the Bio-Oss^®^ group was 1.26 mm at the R1 level and 1.40 mm at the R2 level. After analyzing the initial buccal bone of the groups, it was found to be thinner in the Bio-Oss^®^ group than in the AMDG group (the average value of buccal bone thickness was 1.40 mm and 1.50 mm, respectively). Consequently, the results of this study seemed to be compatible with those found in the literature.

As already mentioned earlier, implant position in the alveolar socket is deemed one of the main factors that affect the extent of buccal bone resorption. Implants with improper positioning have encountered significant buccal bone loss. Moreover, the placement of implants in a position that appeared to be deviated more buccally led to a higher chance of resorption in the buccal bone accompanied by osseous dehiscence [34]. An in vivo study on dogs showed less horizontal bone loss around lingually positioned implants compared to those placed in the middle of the buccolingual alveolar bone ridge [35]. Another clinical trial on humans demonstrated less mid-buccal gingival recession in palatally positioned implants as compared to buccally positioned ones [36]. In the present study, drilling was carried out in a way to create an osteotomy that serves to retain the implant from the apical and palatal walls during implant installation, thus acquiring the primary stability of the implant. Such positioning is recommended as it also protects the buccal bone from resorption and obtains a more favorable 3D positioning of the prosthetic superstructure.

The distinction between standard CT and CBCT images was essential to achieve a superior long-term follow-up. The linear measurement accuracy between the two modalities was of negligible difference statistically. The CBCT machinery production of high-detailed images confronted with low radiation granted a noticeable advancement in the non-invasive assessment of the hard tissue healing around dental fixtures [37]. In order to monitor the effectiveness of both grafts and evaluate the thickness of the buccal bone changes, only three CBCT-scanned patients were selected for the present study, and linear measurements were collected from these images accordingly.

Technical complications related to implant-supported prostheses are commonly encountered nowadays, such as abutment screw loosening and/or fracture, ceramic chipping or fracture, and retention loss of the prosthesis. Screw loosening, for instance, has a high incidence rate (5-year rate: 3.6–10.8%), which in turn leads to the micro-movement of the restoration and subsequently results in peri-implant diseases [38]. In this study, the prosthetic complication rate recorded was 11.8% (*n* = 13/100) with no significant differences detected between both groups. The types of complications observed were ceramic chipping (*n* = 5), screw loosening (*n* = 3), screw fracture (*n* = 1), and decementation (*n* = 4). It was noticed that all the encountered complications were reported only in patients who suffered from bruxism. Consequently, essential precautions were hence reinforced following the treatment of such complications. 

With regard to the esthetic prospects of the implant-related treatment, the PES data were incorporated in this study. Despite the studies that reported the limitation of socket bone grafting on the reduction in bone resorption [13], it still does play a role in maintaining the buccolingual dimension and preventing soft tissue collapse. The average PESs calculated in the present study were 7.96 in the test group and 7.70 in the control group, with no significant difference observed. Such data are considered lower than those reported in several studies published in the literature [39]. Such a difference may be attributed to the protocol of the conventional loading adopted or the lack of any accompanied connective-tissue-grafting procedures performed in the patients of this study. It has been suggested that the preservation of the tissue can be enhanced by the immediate interim restoration, which represents mechanical support for the original gingival margin and interdental papillae [40]. Saito et al. supported this concept as the immediate interim restoration was suggested to have the potential capability to promote implant soft tissue healing and diminish the change in the buccolingual ridge dimension by acting as platform support for the soft tissue [41].

## 5. Conclusions

The present study showed that immediate implantation in a fresh extraction socket accompanied by a simultaneous autogenous mineralized dentin graft or xenogeneic graft material augmentation granted similar outcomes, and both materials were beneficial and advantageous. Based on the clinical and radiological analysis of this study, the autogenous dentin graft showed efficiency, which was relatively comparable to that of the xenogeneic bone graft. Further studies with a larger sample size are required to confirm the presented data.

## Figures and Tables

**Table 1:** Comparison of gender, age, and operation area data of patients.

Variables		AMDG (*n*:57)		Bio-Oss^®^ (*n*:53)		*p*
		*n*	%	*n*	%	
Sex	Female	25	43.9	28	52.8	0.453
	Male	32	56.1	25	47.2	
Location	Anterior	51	89.5	47	88.7	1.000
	Posterior	6	10.5	6	11.3	
		Ave. ± SD (Min–Max)		Ave. ± SD (Min–Max)		
Age ^t^		45.42 ± 9.86 (24–63)		40.28 ± 11.69 (20–63)		0.014 *

* *p* < 0.05, χ2: Chi-square test (Categoric data), ^t^: Independent Sample *t*-test, Ave: average variance extracted. Min: Minimum, Max: Maximum.

**Table 2:** Comparison of buccal bone width, horizontal gap, prosthetic complications, and PES data of patients.

Variables		AMDG (*n*:57)		Bio-Oss^®^ (*n*:53)		*p*
		n	%	n	%	
Prosthetic complication	Yes	50	87.7	47	88.7	1.000
	None	7	12.3	6	11.3	
		Ave. ± SD		Ave. ± SD		*p*
T0 Buccal bone width ^t^		1.34 ± 0.25		1.26 ± 0.21		0.078
T1 Horizontal gap ^t^		2.92 ± 0.34		2.88 ± 0.41		0.515
T2 Buccal bone width ^t^		3.99 ± 0.42		3.85 ± 0.42		0.081
R1 (1 mm) (%) ^z^		6.39 ± 3.78		6.99 ± 5.01		0.078
T0 Buccal bone width ^t^		1.50 ± 0.24		1.40 ± 0.23		0.036 *
T1 Horizontal gap ^t^		2.69 ± 0.34		2.67 ± 0.40		0.763
T2 Buccal bone width ^t^		3.96 ± 0.45		3.78 ± 0.34		0.022 *
R2 (5 mm) (%) ^z^		5.46 ± 4.98		6.77 ± 7.60		0.018 *
PES ^t^		7.96 ± 1.99		7.70 ± 1.88		0.472

* *p* < 0.05, χ2: Chi-square test (Categoric data), ^t^: Independent Sample *t*-test, ^z^: Mann–Whitney U test, Ave: average variance extracted, T0: buccal bone width prior to bone grafting in mm, T1: horizontal gap in mm, T2: buccal bone width 1 year postoperatively, R1: bone graft resorption at 1mm apical to implant collar, R2: bone graft resorption at 5 mm apical to implant collar, PES: Pink Esthetic Score.

**Table 3:** Comparison of buccal bone width, horizontal gap, prosthetic complications, and PES findings based on bone graft type and operation area.

Variables	Location	AMDG (*n*:57)	Bio-Oss^®^ (*n*:53)	
		Ave. ± SD	Ave. ± SD	*p*1
T0 Buccal bone width ^t^	Anterior	1.28 ± 0.19	1.20 ± 0.15	0.029 *
	Posterior	1.82 ± 0.19	1.68 ± 0.08	0.148
	*p*2	0.000 **	0.000 **	
T1 Horizontal gap ^t^	Anterior	2.90 ± 0.30	2.93 ± 0.41	0.681
	Posterior	3.13 ± 0.56	2.47 ± 0.21	0.022 *
	*p*2	0.107	0.009 **	
T2 Buccal bone width ^t^	Anterior	3.90 ± 0,35	3.82 ± 0.44	0.328
	Posterior	4.72 ± 0.28	4.02 ± 0.22	0.001 **
	*p*2	0.000 **	0.296	
R1 (1 mm) (%) ^z^	Anterior	6.61 ± 3.85	7.46 ± 5.12	0.025 *
	Posterior	4.58 ± 2.71	3.29 ± 1.16	1.000
	*p*2	0.131	0.007 **	
T0 Buccal bone width ^t^	Anterior	1.44 ± 0.19	1.35 ± 0.19	0.014 *
	Posterior	1.95 ± 0.20	1.80 ± 0.17	0.186
	*p*2	0.000 **	0.000 **	
T1 Horizontal gap ^t^	Anterior	2.66 ± 0.31	2.73 ± 0.38	0.372
	Posterior	2.92 ± 0.55	2.22 ± 0.14	0.013 *
	*p*2	0.087	0.002 **	
T2 Buccal bone width ^t^	Anterior	3.86 ± 0.35	3.76 ± 0.35	0.127
	Posterior	4.75 ± 0.43	3.97 ± 0.23	0.003 **
	*p*2	0.000 **	0.160	
R2 (5 mm) (%) ^z^	Anterior	5.82 ± 5.14	7.49 ± 7,72	0.007 **
	Posterior	2.45 ± 1.08	1.16 ± 3.15	0.261
	*p*2	0.000 **	0.001 **	
PES ^t^	Anterior	7.98 ± 1.97	7.51 ± 1.83	0.226
	Posterior	7.83 ± 2.32	9.17 ± 1.72	0.284
	*p*2	0.866	0.041 **	

* *p* < 0.05, ** *p* < 0.01, χ2: Chi-square test (Categoric data), ^t^: Independent Sample *t*-test, ^z^: Mann–Whitney U test.

**Table 4:** The prosthetic complication data distribution according to bone graft type and region of operation.

Variables		AMDG (*n*:57)		Bio-Oss^®^ (*n*:53)		*p*
		Number	%	Number	%	
Anterior	Yes	48	94.1	44	93.6	1.000
	None	3	5.9	3	6.4	
Posterior	Yes	2	33.3	3	50.0	1.000
	None	4	66.7	3	50.0	

χ2: Chi-square test (Categoric data).

## Data Availability

The original contributions presented in this study are included in the article; further inquiries can be directed to the corresponding author.
